# Effect of Yeast Inoculation on the Bacterial Community Structure in Reduced-Salt Harbin Dry Sausages: A Perspective of Fungi–Bacteria Interactions

**DOI:** 10.3390/foods13020307

**Published:** 2024-01-18

**Authors:** Yumeng Sui, Xiangao Li, Yuan Gao, Baohua Kong, Yitong Jiang, Qian Chen

**Affiliations:** 1 College of Food Science, Northeast Agricultural University, Harbin 150030, China; suiyumenglz@126.com (Y.S.); lixiangao67@163.com (X.L.); gaoyuan4502@163.com (Y.G.); kongbh@163.com (B.K.); 2 Department of Nutrition and Health, China Agricultural University, Beijing 100193, China

**Keywords:** reduced-salt dry sausage, yeast inoculation, bacterial composition, high-throughput sequencing

## Abstract

Yeast strains are promising starters to compensate for the flavor deficiencies of reduced-salt dry sausages, but their influence on the bacterial community’s structure has not yet been clarified. In this study, the effect of separately inoculating *Pichia kudriavzevii* MDJ1 (Pk) and *Debaryomyces hansenii* HRB3 (Dh) on the bacterial community structure in reduced-salt dry sausage was investigated. The results demonstrated that the inoculation of two yeast strains significantly reduced the pH, and enhanced the total acid content, lactic acid bacteria (LAB) counts, and total bacterial counts of reduced-salt sausages after a 12-day fermentation (*p* < 0.05). Furthermore, high-throughput sequencing results elucidated that the inoculation of yeast strains significantly affected the bacterial composition of the dry sausages. Especially, the relative abundance of bacteria at the firmicute level in the Pk and Dh treatments exhibited a significant increase of 83.22% and 82.19%, respectively, compared to the noninoculated reduced-salt dry sausage treatment (Cr). The relative abundance of *Latilactobacillus*, especially *L. sakei* (0.46%, 2.80%, 65.88%, and 33.41% for the traditional dry sausage (Ct), Cr, Pk, and Dh treatments, respectively), increased significantly in the reduced-salt sausages inoculated with two yeast strains. Our work demonstrates the dynamic changes in the bacterial composition of reduced-salt sausages inoculated with different yeast strains, which could provide the foundation for the in-depth study of fungi–bacteria interactions in fermented foods.

## 1. Introduction

As traditional fermented meat products in northeastern China, Harbin dry sausages are widely preferred by consumers for their unique taste and odor. These sausages are generally manufactured by the fermentation of a mixture of minced lean pork, pork back fat, salt, nitrite, and mixed spices for 9–15 days [1]. Salt is an important additive that provides characteristic flavor and texture and inhibits microbial growth in dry sausages [2,3]. During the preparation of dry sausages, 2.5% NaCl is added to the sausage; however, the level increases to 3.6–5% after the water loss during fermentation [4]. Some studies have shown that meat products are the key source of sodium intake in the daily diet, contributing approximately 20–30% of the total daily salt intake [2]. The World Health Organization (WHO) suggests a maximum daily salt intake of 5 g NaCl/day for adults (about 2 g sodium/day), while, in reality, most people consume 9–12 g/day on average [5]. Excessive salt intake has been linked to various diseases, such as potentially increasing the risk of cardiovascular diseases, hypertension, bone diseases, and kidney diseases [6,7]. General concern about dietary sodium intake has made the development of reduced-salt dry sausages an inevitable trend in the future food industry.

The meat industry has used a variety of strategies to reduce sodium content in meat products [8]. Directly reducing the addition of salt based on product acceptability is more in line with the clean label concept of “minimizing artificial ingredients” and is, therefore, more widely used, but at the same time, it leads to quality and safety issues that should also be considered [9]. The compensatory effects of appropriate starter cultures (e.g., lactic acid bacteria [LAB] and yeast) on the quality and flavor of reduced-salt products have been demonstrated in a variety of food systems, such as reduced-salt sourdough [10], fermented common carp [11], fermented sausages [12,13], and soy sauce [14]. These studies also contribute to a theoretical foundation for further reductions in salt addition.

In our previous studies, we obtained some LAB and yeast strains that were available as starter cultures to offset the taste defects and enhance the flavor of the reduced-salt dry sausages [12,13]. The compensatory effects on taste are mainly due to the accumulation of taste compounds (such as free amino acids and organic acids), promoted by inoculating starter cultures [11]. The improvement in odor is mainly ascribed to the accumulation of the volatiles originating from microbial metabolic pathways, such as acetic acid, 3-hydroxy-2-butanone, ethyl butyrate, 2,3-butanediol, ethyl acetate, and ethanol [15,16]. In the process of food fermentation, a complex microbial ecosystem plays a critical part in quality and flavor enhancements. Notably, the interaction of bacteria (represented by LAB) and fungi (represented by yeast) is considered to facilitate their metabolism [17]. Liu et al. [18] found that yeast and LAB could exchange metabolites in naturally fermented liquor, promoting the production of sulfur-containing compounds and thus promoting the formation of fermentation aroma. Gerardi et al. [19] used *Latilactobacillus plantarum* and *Saccharomyces cerevisiae* to ferment *Prunus mahaleb* fruit, revealing that LAB possess the ability to metabolize malic acid (generated through the metabolism of raw materials and yeast) into lactic acid, thereby mitigating the sour sensation associated with fermented fruit. Huang et al. [20] demonstrated that the incorporation of LAB and *Kluyveromyces marxianus* fermentation in goat milk effectively mitigated the fishy odor associated with improper handling during storage and processing. To date, the effects of fungi inoculation on the bacterial composition and metabolism in dry sausage are seldom clear, especially in reduced-salt meat products.

Therefore, two yeast strains (*Pichia kudriavzevii* MDJ1 and *Debaryomyces hansenii* HRB3) isolated from dry sausages were used in this study, which had exhibited excellent flavor compensation in our previous studies [13]. In this study, we monitored the effects of *P. kudriavzevii* MDJ1 and *D. hansenii* HRB3 on the pH value, total acid content, total bacterial counts, yeast counts, and LAB counts of reduced-salt sausages during fermentation. Meanwhile, the bacterial diversities were investigated using high-throughput sequencing technology to determine the effect of yeast on bacterial composition. This study will establish a theoretical foundation for subsequent investigations into the interaction mechanism between yeast and LAB in fermented meats.

## 2. Materials and Methods

### 2.1. Preparation of Yeast Strains

*P. kudriavzevii* MDJ1 and *D. hansenii* HRB3, the dominant fungi strains, were obtained from dry sausages in Northeast China and identified using a sequence analysis of the rRNA internal transcribed spacer (ITS) [21]. The selected yeast strains have proven to have good technological properties and the ability to improve the flavor profiles of reduced-salt dry sausages [13,21]. These two yeast strains were inoculated in yeast extract peptone dextrose (YPD) medium for two successive incubations of 12 h at 28 °C. The cells were harvested by centrifugating at 6000× *g* for 10 min, and then washed twice with sterile saline. Lastly, the cells were resuspended in sterile saline.

### 2.2. Preparation of Harbin Dry Sausages

**Figure 1:**
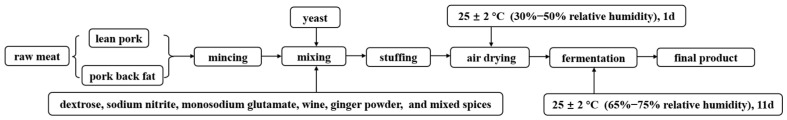
The process flowchart of dry sausage production.

Three independent batches of dry sausages were prepared and a total of four treatments of dry sausages were prepared in each batch (3 batches × 4 treatments). The fresh lean pork and pork back fat used as ingredients were purchased from the fresh market in Harbin, Heilongjiang, China. The dry sausages were prepared based on the methods of Qin et al. [22] with some modifications. Lean pork (1800 g) and pork back fat (200 g) was minced through a 1.5-cm plate with following additives: dextrose (6 g), sodium nitrite (0.18 g), monosodium glutamate (6 g), wine (20 g), ginger powder (10 g), and mixed spices (16 g). The mixed spices comprised *Pericarpium zanthoxyli*, *Angelica dahurica*, *Cinnamomum cassia* Presl, *Syringa oblata* Lindl, *Foeniculum vulgare* Mill, *Amomum cardamon*, *Illicium verum* Hook. F., *Amomum villosum*, and *Piper nigrum* L. The flowchart of dry sausage production is shown in Figure 1. The ingredients were thoroughly mixed and stuffed into natural porcine casings, resulting in each sausage having a length of approximately 20 cm and a diameter of 2.5 cm. Four treatments were manufactured: traditional dry sausage (Ct) and reduced-salt dry sausages (Cr) containing 2.5% and 1.75% NaCl, respectively, were considered as the controls. Pk and Dh treatments (containing 1.75% NaCl) were inoculated with *P. kudriavzevii* MDJ1 and *D. hansenii* HRB3, respectively. First, yeast in the logarithmic growth phase was separately centrifuged at 6000× *g* for 15 min at 4 °C. Then, the resuspended yeast cell pellets were washed twice with sterile saline. Finally, the suspended yeast concentration was adjusted by an OD_600_/stain count standard curve, ultimately ensuring that the yeast concentration inoculated into raw meat was 6 log CFU/g. The sausages were naturally air-dried for 1 day at 25 ± 2 °C (30–50% relative humidity) before being put into the incubator (Shanghai Zhichu Instrument Co., Shanghai, China) for 11 days at the same temperature (65–75% relative humidity). A total of 16 dry sausages (4 treatments × 4 sausages [each treatment]) in each batch were sampled at each time point (day 0, 4, 8, and 12) to determinate pH and total acid content. Additionally, four sausage samples from each treatment were sampled on days 0 and 12 for the bacterial diversity analysis.

### 2.3. Determination of pH and Total Acid Content of Dry Sausages

Ten grams of chopped sausages was homogenized with 90 mL of distilled water [23], and the pH of homogenizate was measured by a pH meter (2018C132-1, Sardonis Scientific Instruments Co., Shanghai, China). The total acid content was determined based on acid-base titration according to the method of Zhang et al. [24].

### 2.4. Determination of Total Bacterial, Yeast, and LAB Counts

According to the method Omer et al. [25] and Niamah [26], 10 g of chopped sausage samples was mixed with 90 mL of sterile saline. The mixtures were shaken with an oscillator (HY-2, Guohua Electric Co., Changzhou, China) for 30 min. Serial decimal dilutions were performed on the sausage sample mixtures. The total bacterial counts were determined using Plate Count agar (PCA; Qingdao Haibo Biotechnology Co., Qingdao, China) after incubation at 37 °C for 48 h. Cycloheximide (0.1 g/L) was also included to suppress the growth of fungi and promote the selectivity of the PCA media. Yeast counts were determined using Yeast Extract Peptone Dextrose agar (YPD; Qingdao Haibo Biotechnology Co., Qingdao, China) containing chloramphenicol after incubation at 30 °C for 48 h. The LAB counts were determined using Man–Rogosa–Sharpe agar (MRS; Qingdao Haibo Biotechnology Co., Qingdao, China) containing cycloheximide (Aladdin Biochemical Technology Co., Shanghai, China) after incubation at 37 °C for 48 h.

### 2.5. Bacterial Diversity Analysis

#### 2.5.1. DNA Extractions

The extraction of bacterial DNA from sausages was carried out according to the method of Xiao et al. [27]. The DNA quality was monitored by 1% agarose gel electrophoresis. The concentration and purity of DNA extract was determined by NanoDrop 2000 spectrophotometer (Thermo Scientific, Wilmington, DC, USA).

#### 2.5.2. Amplification and Sequencing of 16S rRNA

The V1–V9 hypervariable regions of 16S rRNA genes were amplified by forward and reverse primers, as described by Hu et al. [28]. The PCR amplification was run following the method of Wang et al. [29]. After performing PCR extraction, purification, and library preparation for sequencing, the library was sequenced on a Pacific Sequel platform (PacBio) by Novogene Co. (Beijing, China, https://www.novogene.com/, accessed on 13 September 2023).

#### 2.5.3. Data Processing and Bioinformatic Analysis

The raw 16S rRNA gene sequences were first processed by the PacBio single-molecule real-time sequencing (SMRT) portal for more than three filtering cycles to ensure a minimum prediction accuracy of 90%. Subsequently, the generated files were trimmed to the amplicon size (800–2000 bp). The reads were assigned to samples and truncated by cutting off the barcode and primer sequence according to the method of Haas et al. [30] and Hu et al. [28] to obtain clean reads. All clean reads were clustered using UPARSE software. Based on their similarity (default ≥ 97%), these sequences were assigned to operational taxonomic units (OTUs), in which representative sequences for each OTU were screened for further annotation [24]. Species annotation analysis (threshold 0.8–1) of representative sequences of OTUs was performed using the Mothur method with the SSU rRNA database from SILVA to obtain taxonomic information and community composition of the samples at each taxonomic level. The alpha diversity indices were calculated by QIIME software (Version 1.9.1). For beta diversity, the QIIME software (Version 1.9.1) was used to calculate UniFrac distance, and R software (Version 2.15.3) was used to draw the principal coordinates analysis (PcoA) plots.

### 2.6. Statistical Analysis

A statistical analysis of the data was performed using the Statistix 8.1 software (Analytical Software, St. Paul, MN, USA). The results were expressed as mean values ± standard errors (SE). In this study, the analysis of variance (ANOVA) was used in conjunction with Tukey’s multiple comparison test to identify significant differences between samples (*p* < 0.05). The pH, total acid content, LAB count, and yeast count of the sausages were analyzed by a mixed model. In this model, each replicate was included as a random term, and different treatments (Ct, Cr, Pk, and Dh) and fermentation times (0, 4, 8, and 12 days) were included as fixed terms.

## 3. Results and Discussion 

### 3.1. pH and Total Acid Content Analysis

**Figure 2:**
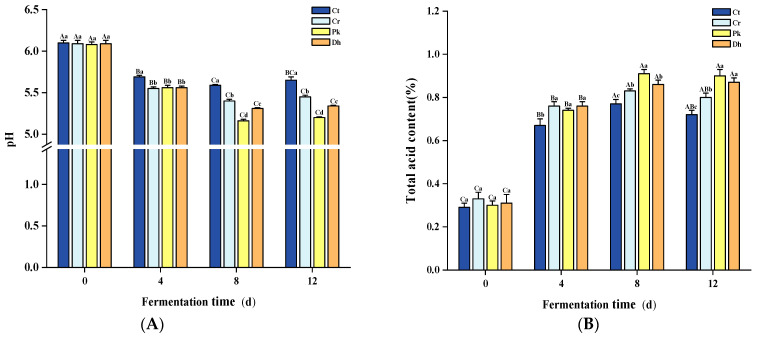
Changes in pH (**A**) and total acid content (**B**) of the control and reduced-salt Harbin dry sausages non-inoculated and inoculated with different yeast strains during fermentation. ^A–C^ Different uppercase letters indicate significant differences among the different fermentation times for the same treatment; ^a–d^ Different lowercase letters indicate significant differences among the different treatments for the same fermentation time (*p* < 0.05). Ct: 2.50% NaCl; Cr: 1.75% NaCl; Pk: 1.75% NaCl + *P. kudriavzevii* MDJ1; Dh: 1.75% NaCl + *D. hansenii* HBR3.

The pH and total acid content are typical indicators of fermented meat products, mainly reflecting the organic acid content of the system and fermentation process [31,32]. The different treatments and fermentation times had significant effects on the pH and total acid content (*p* < 0.05). A significant decrease in pH was observed in all dry sausages (Figure 2A; *p* < 0.05), from approximately 6.09 (0 days) to 5.07–5.59 (8 days), due to the rapid multiplication of LAB, as described in our previous study [13]. During this time, these LAB metabolized the added carbohydrates in the sausage preparation to produce lactic acid [33]. Meanwhile, the trend of total acid content showed a rapid increase (Figure 2B). Subsequently, in the later stages of fermentation (8–12 days), there was no fluctuation in pH and total acid content in dry sausages (*p* > 0.05), which could be associated with the buffering effect on organic acids by amino groups produced by protein degradation [34]. In terms of interaction between the treatments and fermentation times, statistically significant differences were observed in the pH and total acid content (*p* < 0.05).

The sausages with the highest salt addition, the Ct treatment, had the highest pH throughout the fermentation period, which is in accordance with the results of Hu et al. [35]. This may be because the inclusion of more salt can extract more soluble proteins from muscle cells and increase the buffering effect [36]. Furthermore, a high salt content inhibits microorganism growth and restrains carbohydrate metabolism [37]. The significant differences in pH and total acid content among the reduced-salt treatments were mainly observed at 8–12 days. On day 12, the pH of the Pk and Dh treatments was lower than that of the Cr treatment, and the total acid content showed the opposite trend (*p* < 0.05). This difference may be due to the combined action of yeast and LAB: the hydrolysis of sucrose into glucose and fructose by yeasts provides a source of carbohydrates that is more readily metabolized by LAB [38]. In particular, the dry sausages inoculated with *P. kudriavzevii* MDJ1 showed the lowest pH and the highest total acid content at the late fermentation stage (8–12 days). This could be attributed to the potentially higher bioavailability of *P. kudriavzevii* MDJ1 metabolites for LAB compared to *D. hansenii* HRB3 metabolites, thereby promoting the growth of LAB and subsequent acid production [18]. Similarly, the inoculation of yeast strains had significant effects on the total acid content of the sausages on day 12 (*p* < 0.05).

### 3.2. Total Bacterial, Yeast, and LAB Counts Analysis

**Figure 3:**
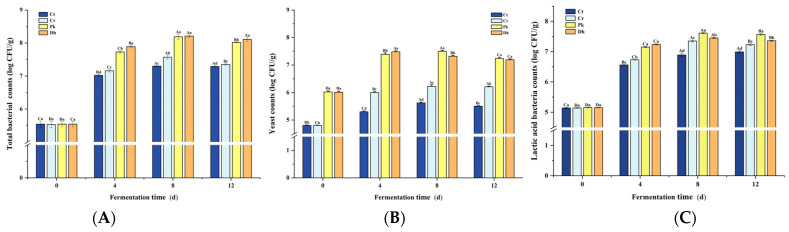
Changes in total bacterial counts (**A**), yeast counts (**B**), and lactic acid bacteria counts (**C**) of the control and reduced-salt Harbin dry sausages, non-inoculated and inoculated with different yeast strains during fermentation. ^A–D^ Different uppercase letters indicate significant differences among the different fermentation times for the same treatment; ^a–d^ different lowercase letters indicate significant differences among the different treatments for the same fermentation time (*p* < 0.05). Ct: 2.50% NaCl; Cr: 1.75% NaCl; Pk: 1.75% NaCl + *P. kudriavzevii* MDJ1; Dh: 1.75% NaCl + *D. hansenii* HBR3.

Total bacterial, yeast, and LAB counts significantly increased (*p* < 0.05) from day 0 to day 4, generally peaking on day 4 or 8 (Figure 3). The total bacterial, yeast, and LAB counts were affected not only by the different strain inoculations but also by the fermentation time and their interaction (*p* < 0.05). The total bacterial, yeast, and LAB counts in the Cr treatment were consistently lower than those in the inoculated treatments during fermentation (*p* < 0.05). Throughout the fermentation process, reduced-salt dry sausages consistently exhibited higher total bacterial counts than traditional dry sausages (Figure 3A). At the end of fermentation (12 d), the highest total bacterial count was observed in the Dh treatment (8.35 log CFU/g) compared to the Pk, Cr, and Ct treatments (8.02, 7.35, and 7.29 log CFU/g, respectively; *p* < 0.05). This result can be attributed to the utilization of metabolites from *P. kudriavzevii* MDJ1 and *D. hansenii* HRB3 by bacteria in the dry sausage system, thereby promoting the growth of bacteria [18]. From day 4 onward, the inoculated treatments had higher yeast counts than the non-inoculated treatments, suggesting that both yeast strains were colonized successfully (Figure 3B; *p* < 0.05). After a 12-day fermentation, the two inoculated treatments displayed the same yeast counts of approximately 7.20 log CFU/g (*p* > 0.05). In addition, the counts of LAB in the inoculated treatments were significantly higher than those in the non-inoculated treatment (Cr) (*p* < 0.05), suggesting that the inoculation of yeast strains may promote the growth of LAB to some extent. The counts of LAB in the treatment inoculated with *P. kudriavzevii* MDJ1 were significantly higher (7.57 log CFU/g) compared to those in the treatment inoculated with *D. hansenii* HBR3 (7.36 log CFU/g), which is consistent with the pH results. This result can be attributed to the production of amino acids and vitamins by yeast, which act as nutritional factors for LAB, thereby promoting the growth of LAB [39]. In fermented foods, the diverse core microbial consortia (such as LAB, yeast, and filamentous fungi) not only interact with the fermentation substrate but also with each other [29], performing diverse biological activities responsible for the texture, flavor, nutrition, and safety of fermented products. As the dominant bacteria and fungi in dry sausages, substantial research has been undertaken on the interaction mechanism between LAB and yeast based on their nutritional metabolites, particularly in products such as sourdough, kefir, and wine [40,41,42]. Generally, the amino acids, carbon dioxide, and vitamins produced by the metabolic pathways in yeast promote the growth of LAB, and LAB can break down lactose into galactose and glucose, providing a carbon source for the yeast [43,44]. In addition, the bacteria and fungi can also interact in other ways, including environmental stress, chemotaxis, direct cell–cell contact, the secretion of quorum-sensing molecules, and gene transfer [45]. However, the strain pair that is involved has a significant impact on the type of interaction [46].

### 3.3. Bacterial Diversity Analysis

#### 3.3.1. Alpha Diversity of the Bacterial Community

The bacterial diversity and community structure of Harbin dry sausages on days 0 and 12 were characterized. Due to the absence of a significant difference in the bacteria composition among the four treatments at the beginning of fermentation (day 0), only one sample was selected as a representative for analysis. As presented in Table 1, the number of high-quality SMRT sequencing reads for the five treatments ranged from 18,966 to 48,460, with an average of 32,611.6 sequences per sample. Good’s coverage was more than 99% for all treatments, suggesting that most bacterial phylotypes were well-detected. The diversity indices (Shannon and Simpson) and the richness indices (Chao 1 and ACE) increased after fermentation in all treatments, indicating that fermentation increased the bacterial diversity. The Cr treatment demonstrated more OTUs and higher diversity and richness indices than the Ct treatment, implying that the low-salt treatment led to a high level of diversity. At the same salt content, the Dh treatment had the highest diversity and richness indices, followed by the Cr and Pk treatments. It is suggested that the inoculation of *D. hansenii* increased the bacterial diversity, which may be related to the fact that the inoculation of yeast promoted the growth of some dominant LAB (such as *Latilactobacillus sakei*, as shown in Figure 5).

**Figure 4:**
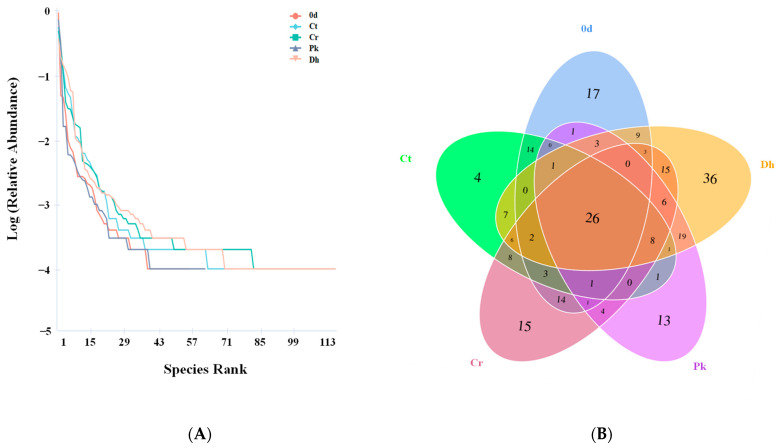
Rank abundance plot (**A**) and Venn diagram (**B**) of the control and reduced-salt Harbin dry sausages non-inoculated and inoculated with different yeast strains on days 0 and 12 based on the OTUs of bacteria. 0 d: the sausages on day 0; Ct: 2.50% NaCl; Cr: 1.75% NaCl; Pk: 1.75% NaCl + *P. kudriavzevii* MDJ1; Dh: 1.75% NaCl + *D. hansenii* HBR3.

Rank abundance curves were plotted based on the level of OTUs to visualize the richness and evenness of species in the treatments (Figure 4A). In the horizontal direction, the Dh treatment had the largest span on the horizontal axis, indicating that this had the highest species richness, followed by the Cr treatment. In the vertical direction, the curves of the Cr and Dh treatments were flatter than those of the other treatments, implying their more uniform species distribution. The Venn diagram was used to estimate the unique and shared OTUs of the bacterial communities of all treatments. As shown in Figure 4B, 95, 82, 112, 85, and 142 OTUs were obtained from the treatment on day 0, and the Ct, Cr, Pk, and Dh treatments on day 12, respectively, among which 26 OTUs were observed to be common in all treatments. Moreover, the sausages inoculated with *D. hansenii* HRB3 showed the highest level of unique OTUs, reaching 36 OTUs, indicating the low-level similarity of bacterial diversity with the other treatments. 

#### 3.3.2. Composition of the Bacterial Community

**Figure 5:**
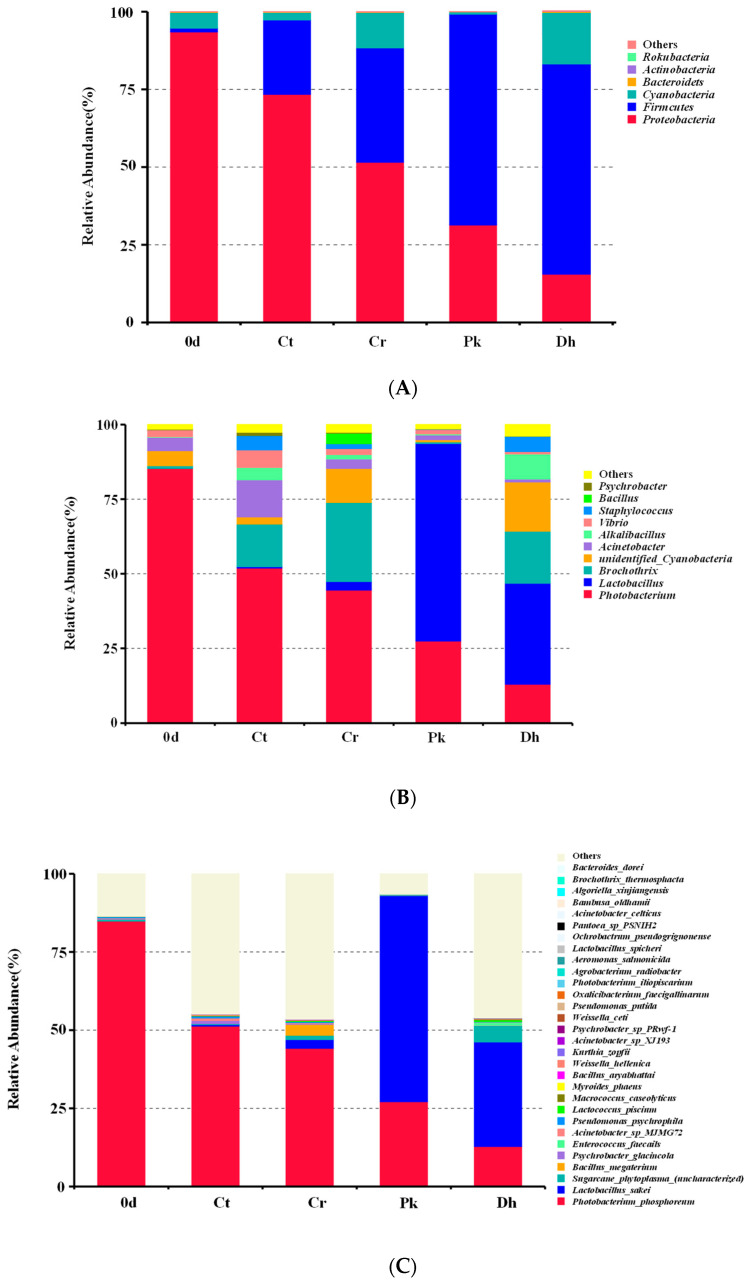
Relative abundance of bacterial compositions at the phylum level (**A**), genus level (**B**), and species level (**C**) of the control and reduced-salt Harbin dry sausages, non-inoculated and inoculated with different yeast strains on days 0 and 12. 0 d: the sausages on day 0; Ct: 2.50% NaCl; Cr: 1.75% NaCl; Pk: 1.75% NaCl + *P. kudriavzevii* MDJ1; Dh: 1.75% NaCl + *D. hansenii* HBR3.

After the classification of the OTUs, a total of six bacterial phyla were identified across all treatments on days 0 and 12, as shown in Figure 5A. *Proteobacteria*, *Firmicutes*, and *Cyanobacteria* were the dominant phyla in all treatments, accounting for over 99% of all OTUs. In addition, *Bacteroidetes*, *Actinobacteria*, and *Rokubacteria*, with relative abundances of less than 1%, were also identified in some treatments. On day 0, the dominant phylum in the dry sausages was *Proteobacteria* (93.51%), followed by *Cyanobacteria* (5.04%) and *Firmicutes* (1.21%). After a 12-day fermentation, *Proteobacteria*, *Cyanobacteria*, and *Firmicutes* remained dominant in all dry sausages. For all treatments, there was a rise in the relative abundance of *Firmicutes* and a fall in the relative abundance of *Proteobacteria* throughout the fermentation period. The same phenomenon was observed by Zhang et al. [24] in the fermentation process of low-salt fermented sausages. It is reported that *Firmicutes* is closely associated with carbohydrate metabolism, and *Proteobacteria* is associated with amino acid and lipid metabolism [47]. The abundances of *Firmicutes* and *Cyanobacteria* in the Cr treatment (36.78% and 11.40%, respectively) were higher than those in the Ct treatment (23.92% and 2.42%, respectively), whereas the Ct treatment exhibited a higher abundance of *Proteobacteria* than the Cr treatment. The inoculation of *P. kudriavzevii* MDJ1 and *D. hansenii* HRB3 both enhanced the relative abundance of *Firmicutes* (67.91% and 67.52%, respectively) and reduced the relative abundance of *Proteobacteria* (31.35% and 15.56%, respectively).

The relative abundance of bacteria in dry sausages at the genus level showed that *Photobacterium*, *Latilactobacillus*, *Brochothrix*, unidentified *Cyanobacteria*, and *Acinetobacter* were relatively abundant (Figure 5B). In the treatment on day 0, *Photobacterium* was the most frequently represented genus (85.27%), followed by unidentified *Cyanobacteria* (5.04%), *Acinetobacter* (4.65%), and *Vibrio* (2.30%). After a 12-day fermentation, *Latilactobacillus*, *Brochothrix*, *Acinetobacter*, *Alkalibacillus*, and *Staphylococcus* also became dominant in specific treatments. It was noteworthy that the relative abundance of *Latilactobacillus* noticeably increased when *P. kudriavzevii* MDJ1 and *D. hansenii* HRB3 were inoculated, which increased by 63.32% and 31.00% compared to the non-inoculated treatment (Cr treatment), respectively, which was consistent with the previous observation that the growth of LAB depends on the metabolites (mainly nitrogenous compounds) produced by yeast [44,48]. The result could be attributed to the protective effect of exopolysaccharides produced by yeast on LAB against various stressors [49]. LAB are dominant bacteria in various fermented meat products (such as fermented dry-cured beef, fermented jerky, and fermented sausages), with quality-enhancing effects [50,51,52]. Some LAB possess protease and lipase activities that contribute to the formation of peptides, free amino acids, and free fatty acids [1]. Furthermore, in our previous study, we verified the flavor compensation role of *P. kudriavzevii* MDJ1 and *D. hansenii* HRB3 in reduced-salt dry sausages, resulting in an improvement in the contents of certain volatile compounds [13]. Therefore, an increase in the relative abundance of LAB caused by the inoculation of yeasts may be a crucial pathway for influencing the flavor characteristics of reduced-salt dry sausages. The increase in the abundance of *Latilactobacillus* was accompanied by a decrease in some spoilage-related microorganisms, such as *Photobacterium*, *Brochothrix*, *Acinetobacter*, *Vibrio*, *Bacillus*, and *Psychrobacter*. These spoilage bacteria have been proven to be associated with meat component degradation, slime formation, and the undesirable odor and appearance of meat [53,54,55]. This phenomenon may be due to the fact that some LAB can produce antimicrobial metabolites to inhibit the growth of spoilage bacteria during sausage fermentation [56]. Similarly, the above-mentioned spoilage microorganisms showed a higher abundance in the Cr treatment compared to the Ct treatment, indicating that the salt reduction was less effective in inhibiting spoilage microorganisms. Moreover, Fadahunsi and Olubodun [57] reported that some yeasts could secrete inhibitory compounds (e.g., diacetyl, organic acids, and antibiotic factors) that inhibit foodborne pathogens and food spoilage microorganisms, explaining the lower abundance of spoilage bacteria in the inoculated treatments than in the Cr treatment. In addition, relatively high levels of *Staphylococcus* were also detected in the Ct (4.79%), Cr (1.58%), and Dh (5.07%) treatments after fermentation. Many enzyme activities have been detected in *Staphylococcus* isolated from fermented meat products, including proteolytic, lipolytic, nitrate reductase, decarboxylase, and antioxidant activities, which distinctly affect the quality and flavor of the products [58].

As depicted in Figure 5C, *Photobacterium phosphoreum* (12.79–84.34%), *L. sakei* (0–65.75%), *Sugarcane phytoplasma* (0.12–5.27%), *Bacillus megaterium* (0–3.43%), *Psychrobacter glacincola* (0.06–1.04%), *Enterococcus faecalis* (0–0.95%), *Acinetobacter* sp. MJMG72 (0.08–0.81%), *Pseudomonas psychrophile* (0.03–0.57%), *Lactococcus piscium* (0.04–0.55%), and *Macrococcus caseolyticus* (0–0.22%) were the top nine identified species. From the composition of bacteria at the species level, the fermentation time, the salt content, and the inoculation of yeast strains could lead to remarkable changes in the bacterial composition of dry sausages. *P. phosphoreum*, often found in spoiled fish, was the dominant bacterial species in the treatment on day 0 (84.34%), and the Ct (50.78%) and Cr (43.75%) treatments on day 12 [59]. *L. sakei* was detected in the sausages fermented for 12 days, and its relative abundance was highest in the Pk treatment (65.74%), followed by the Dh (33.37%) and Cr (2.75%) treatments. The results indicated that *P. kudriavzevii* MDJ1 exhibited a significantly higher capacity to promote the abundance of *L. sakei* compared to *D. hansenii* HRB3. *L. sakei* commonly exists in fermented sausages [33], kimchi [60], and sourdough [61] and is well-adapted to high acidity levels due to the ammonia-releasing and energy-providing arginine deiminase pathway [62]. It mainly promotes acidification, inhibiting microbial growth and decomposing carbohydrate and amino acids to produce volatile compounds [32,63].

### 3.4. Beta Diversity of the Bacterial Community

**Figure 6:**
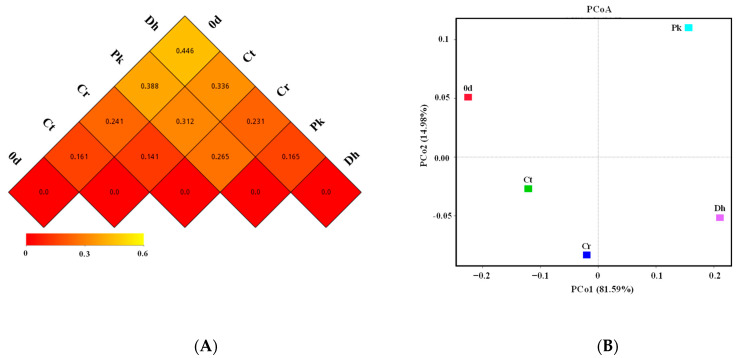
Beta diversity index heatmap (**A**) and principal coordinate analysis (**B**) of the control and reduced-salt Harbin dry sausages, non-inoculated and inoculated with different yeast strains on days 0 and 12. 0 d: the sausages on day 0; Ct: 2.50% NaCl; Cr: 1.75% NaCl; Pk: 1.75% NaCl + *P. kudriavzevii* MDJ1; Dh: 1.75% NaCl + *D. hansenii* HBR3.

To further reveal the differences in the bacterial community structures of all treatments, a beta diversity analysis was performed. The dissimilarity coefficients based on the weighted UniFrac distance between treatments were reflected (Figure 6A), where a smaller coefficient implies a more similar bacterial community structure. Among all treatments, the Ct treatment had the smallest dissimilarity coefficient with the Cr treatment (0.141), followed by the treatment on day 0 (0.161), the Pk treatment on day 12 (0.312), and the Dh treatment on day 12 (0.336). The inoculation of *P. kudriavzevii* MDJ1 and *D. hansenii* HRB3 had a greater effect on the bacterial community structure of dry sausages compared to the salt reduction. In the inoculated dry sausages, the bacterial structure of the Pk treatment was more similar to the traditional dry sausages (Ct treatment), whereas the Dh treatment was more similar to the reduced-salt dry sausages (Cr treatment).

PCoA was used to identify the clustering pattern of the bacterial communities in Harbin dry sausages (Figure 6B). The first two principal coordinates (PCo1 and PCo2) explained 96.57% of the total variance (81.59% and 14.98%, respectively) in the bacterial assemblages. The Ct and Cr treatments clustered close to each other in the third quadrant, whereas the other treatments were distinctly separated from each other. This result again illustrates the significant effects of yeast inoculation on the bacterial composition of dry sausages.

## 4. Conclusions

The present results showed that the inoculation of *P. kudriavzevii* MDJ1 and *D. hansenii* HRB3 significantly promoted the growth of bacteria and LAB and increased the relative abundance of *Latilactobacillus*, especially *L. sakei*, in reduced-salt dry sausages. Accordingly, the inoculated dry sausages showed a reduced pH and increased total acid content. A beta diversity analysis indicated that the inoculation of yeast strains significantly influenced the bacterial composition structure of dry sausages. This work may help to clarify the interactions between bacterial and fungal communities in fermented dry sausages. Further studies should focus on the interaction mechanism between specific bacterial and fungal strains, providing a theoretical basis for artificially regulating microbial community function.

## Figures and Tables

**Table 1:** The diversity and richness indices of bacteria in the control and reduced-salt Harbin dry sausages, non-inoculated and inoculated with different yeast strains on days 0 and 12.

	0 d	Ct	Cr	Pk	Dh
Total reads	48,460	18,966	23,383	42,345	29,904
Good’s coverage	0.998	0.999	0.998	0.998	0.996
Shannon	1.145	2.599	2.680	1.475	3.092
Simpson	0.285	0.702	0.727	0.495	0.820
Chao 1	75	76.889	114.517	81.714	164
ACE	81.846	81.810	120.716	107.716	183.796

0 d: the sausages on day 0; Ct: 2.50% NaCl; Cr: 1.75% NaCl; Pk: 1.75% NaCl + *P. kudriavzevii* MDJ1; Dh: 1.75% NaCl + *D. hansenii* HBR3.

## Data Availability

Data is contained within the article.
